# Profile and artificial insemination practices of technicians and the artificial insemination success rates in Leyte, Samar, and Biliran, Philippines (2011-2015)

**DOI:** 10.14202/vetworld.2017.181-186

**Published:** 2017-02-13

**Authors:** Adrian P. Ybañez, Rochelle Haidee D. Ybañez, Maxine O. Caindec, Louie V. Mani, Julius V. Abela, Edgar S. Nuñez, Johnson T. Royo, Ivy Fe M. Lopez

**Affiliations:** 1 Department of Biology and Environmental Science, College of Science, University of the Philippines Cebu, Lahug, Cebu City 6000, Philippines; 2 Department of Research, Gullas College of Medicine, University of the Visayas, Banilad, Mandaue City 6014, Cebu, Philippines; 3 Philippine Carabao Center at Visayas State University, Visca, Baybay City 6521-A, Leyte, Philippines

**Keywords:** artificial insemination, artificial insemination technician, Philippines, profile, water buffalo

## Abstract

**Background:**

Artificial insemination (AI) is a reproductive biotechnology that may be influenced by several factors, including the profile of the technicians and the practices used. Assessing technician’s profile and their AI practices can be significant in improving AI success rate.

**Aim:**

This study aimed to know the profile and current practices used by AI technicians (AITs), to determine the success rates of AI in water buffaloes in Leyte, Samar, and Biliran from 2011 to 2015, and to evaluate the possible association between the parameters investigated.

**Materials and Methods:**

A total of 50 AITs from Leyte, Samar and Biliran, Philippines, were interviewed using a fixed questionnaire about their profile and employed AI practices, and 20,455 AI-related records of the Philippine Carabao Center (PCC) at Visayas State University (VSU), Baybay City, Leyte, were screened and analyzed. AI success rates were determined by retrospective analysis of the gathered data. Statistical analysis was performed between the technician profile and practices and the AI success rates.

**Results:**

Results revealed that most of the technicians were male, around 31-40 years old, married, college graduates, working under local government units, had other sources of income, and with 1-5 years of continuous AI practice averaging 51-100 inseminations per year. Most of them attended only one basic training seminar, which was conducted more than 3 years ago in PCC in VSU. AI success rates were recorded highest in 2011 and lowest in 2015. Statistical analyses showed that some technician profile parameters (civil status, average AI per year, and the training center) and several practices (checking of soft cervix, rectal palpation, thawing temperature method, straw cutting method, and semen deposition) might have an influence on the success of AI.

**Conclusion:**

This study documents the first report on AIT’s profile and their employed AI practices and the AI success rates in Leyte, Samar, and Biliran, Philippines. Selected profile parameters and AI practices may influence AI success rates. AITs should perform more AI services and revisit the employed practices.

## Introduction

Artificial insemination (AI) is among the most effective breeding methods that afford widespread propagation of genes carried by superior males [1]. It is more profitable than the natural service method [2]. The success of AI depends on several factors, including the technician and practices employed [3,4]. Assessing technician’s profile and their AI practices can be significant in improving AI success rate.

In the Philippines, the use of AI for livestock started on the establishment of the Philippine Carabao Center (PCC) in 1993 [5]. Water buffaloes (*Bubalus bubalis*) are considered integral to the Philippine agricultural industry. Although these animals are mostly used for draft, its utilization as a food source may have increased, which may have contributed to its population decline. According to the Philippine Bureau of Agricultural Statistics, the total buffalo population in 2004 was 3.28 million, which decreased to 2.96 million in 2011. Despite the Carabao Development Program of PCC [6], the water buffalo population has been declining. In Eastern Visayas Region (EVR), Typhoon Haiyan may have also contributed to its reduction. Recent reports indicated that EVR accounted for 9.7% of the total water buffalo population in the country. To help improve the declining water buffalo population, AI has been practiced in the region. However, on a national scale, the success rates are considered low [7].

While studies on AI appear to be well reported in other countries, reports and studies in the Philippines remain limited or are unpublished and are usually difficult to access. Environment and management [8,9], which have been shown to affect AI, may differ depending on the location, economic condition, and culture. Studying the profile of technicians and practices employed and the success of AI in a selected area will be beneficial not only to the Philippines but also to other countries having similar conditions. Hence, this study was conducted.

## Materials and Methods

### Ethical approval

The study was conducted in accordance with the principles of Helsinki declaration developed by the World Medical Association. Informed consent was obtained from the respondents. No live animal was used in this study.

### Study design, selection of respondents and study area

This study utilized a descriptive-analytical design and was conducted in five areas from the VSU, Philippines (Leyte, Southern Leyte, Biliran, Western Samar and Northern Samar) from March to May 2016. Out of the 250 AI technicians (AITs) (regardless of sex, status, educational attainment, employment, and age) contacted, only 50 were able to complete the questionnaires.

### Survey questionnaire and AI records

The pre-tested, researcher-made, fixed questionnaire composed of two parts. The first part covered the profile of the AITs, including age, sex, civil status, educational attainment, AI training, nature of job assignment, receipt of service fees, other sources of income, nature of income sources, number of years as AIT, average number of AI per year, number of AI trainings attended, level of training attended, place of training, and last AI training attended. The second part included the practices employed. AI success rates (calf drop and the number of animals provided with AI services) were determined based on the available records at the PCC in Visayas State University (VSU), Baybay City, Leyte. A total of 20,455 records were screened: 2011 (2993), 2012 (4120), 2013 (4857), 2014 (4645), and 2015 (3840).

### Data processing and statistical analysis

Data gathered from the questionnaires and AI records were manually tabulated and encoded in Microsoft Excel Office using appropriate variable coding. Encoded data were then imported into statistical software for analysis. Descriptive statistics was employed. Significant differences between technician profile factors and AI success rates were analyzed using one-way ANOVA and independent t-tests.

## Results and Discussion

### AIT profile

Information on profile revealed that most AITs were from Northern Leyte (42%), male (92%), 31-40 years old (36%), and married (78%) (Table-1). These findings were similar to the study of Russi *et al*. [10] where most AITs were also college graduates (58%). Singh and Nanda [11] showed that higher success rates were found in professional degree holders than diploma holders. 30 technicians were working under local government units (LGUs). The majority of the technicians did not receive fees for the AI services rendered since they had other sources of income. The initiative of PCC in 2006 to train farmers to work as village-based AITs was evident with 38% of the technicians being land farmers also. These technician farmers did not receive fees for their services as they were closely associated with their community, implying that interpersonal relationships were more important than receiving remuneration.

**Table-1:** Profile of AITs in Eastern Visayas (n=50).

Parameter	n (%)
Location	
Leyte	21 (42.0)
Southern Leyte	15 (30.0)
Biliran	2 (4.0)
Western Samar	6 (12.0)
Northern Samar	6 (12.0)
Sex	
Male	46 (92.0)
Female	4 (8.0)
Age	
18-30	7 (14.0)
31-40	18 (36.0)
41-50	16 (32.0)
Above 50	9 (18.0)
Range: 23-62, Mean: 40.8, SD: 8.7	
Civil status	
Single	7 (14.0)
Married	39 (78.0)
Widowed	1 (2.0)
Prefer not to say	3 (6.0)
Highest educational attainment	
Elementary graduate	1 (2.0)
High school level	6 (12.0)
High school graduate	6 (12.0)
College level	7 (14.0)
College graduate	29 (58.0)
Post-graduate	1 (2.0)
Nature of job assignment	
LGU	30 (60.0)
Community/village based	15 (30.0)
Prefer not to say	5 (10.0)
Receipt of service fees	
Not at all	26 (52.0)
Always	9 (18.0)
Sometimes	12 (24.0)
Prefer not to say	3 (6.0)
Others source of income	
No	10 (20.0)
Yes	34 (68.0)
Prefer not to say	6 (12.0)
Nature of other income sources	
Land farming	19 (38.0)
Fishing	3 (6.0)
Animal raising	16 (32.0)
Others	3 (6.0)
Number of years as AIT	
<1	3 (6.0)
1-5	26 (52.0)
6-10	10 (20.0)
Above 10	8 (16.0)
Prefer not to say	3 (6.0)
Range: 1 month-18 years, Mean: 5.8, SD: 4.6	
Type of AI practice	
Continuous	43 (86.0)
Discontinuous	5 (10.0)
Prefer not to say	2 (4.0)
Average AI per year	
10-50	18 (36.0)
51-100	20 (40.0)
Above 100	5 (10.0)
Prefer not to say	3 (6.0)
Range: 10-250, Mean: 70.4, SD: 47.9	
Number of AI trainings attended	
1	30 (60.0)
2	12 (24.0)
More than 2	6 (12.0)
Prefer not to say	1 (2.0)
Level of training attended	
Basic	42 (84.0)
Advanced	3 (6.0)
Prefer not to say	5 (10.0)
Place of trainings	
Ubay, Bohol	24 (48.0)
VSU	31 (62.0)
University of the Philippines/Central Luzon State University	2 (4.0)
Others	4 (8.0)
Last AI training attended	
Within 1 year	9 (18.0)
2 years ago	6 (12.0)
3 years ago or more	32 (64.0)
Prefer not to say	3 (6.0)

AIT=Artificial insemination technician, LGU=Local government units, VSU=Visayas State University

The majority of technicians had 1-5 years of continuous AI practice, with an average number of 51-100 inseminations per year. Several studies have shown that experienced technicians can have higher success rates than new technicians [12]. Most technicians had attended only one basic training seminar, conducted more than 3 years ago. Akhtar *et al*. [13] and Boettcher and Perera [14] showed that higher success rates were associated with technicians who attended refresher courses. Majority attended their training at PCC in VSU, probably because this is the training center closest to Leyte, Samar, and Biliran. On the other hand, the number of AI technicians after 10 years of AI practice declined. This may be due in part of the reassignments in the LGU, retirement or shift of career path.

### AI practices

All AITs checked heat before performing AI. Heat was confirmed mostly (92%) through the appearance of clear mucus discharges. The majority (90%) of the AITs checked the time of heat before insemination. Time of response of 70% of AITs was within 12 h after they are informed of the status of the animal. Most (84%) AITs used a thermometer to check the thawing temperature of semen. All the AITs checked thawing time. However, only 22% used a watch to do this and 76% used manual/mental counting. A thawing time of 1-10 s was practiced by 56% of the AITs. Either cutter (54%) or scissors (58%) was used to cut the straw, with about 76% of the AITs cutting the straws straight. Almost all (98%) used gloves and 52% did not reuse them. The majority (66%) used soap as a lubricant, but there were still 16% that did not use any. AITs deposited the semen in the body of the uterus (86%) and the mid-cervix if the standard time of AI has lapsed (44%). The preferred time of insemination was morning (44%). Insemination was done 3 times by 74% of AITs. Almost all (96%) AITs confirmed pregnancy at 3 months after AI (64%). Estrus synchronization was also done by most AITs (86%). All of them educate the farmers on these different profiles (Table-2).

**Table-2:** AI practices employed by technicians in Eastern Visayas (n=50).

Parameter	n (%)
Checking of heat prior to AI	
Yes	50 (100)
No	0 (0)
Method of checking heat	
Vulval swelling	30 (60)
Soft cervix	9 (18)
Rectal palpation	32 (64)
Reddish coloring of vagina	32 (64)
Clear mucus discharge	46 (92)
Others	2 (4)
Pre-AI information gathered	
Time of discharge	23 (46)
Time of heat	45 (90)
Amount of discharge	12 (24)
Species of water buffalo	8 (16)
Time (hours) of response after gathering information	
1-12	35 (70)
13-24	2 (4)
Above 24	3 (6)
Method of checking thawing temperature	
Not at all	2 (4)
Thermometer	42 (84)
Hand	9 (18)
Checking of thawing time	
No	0 (0)
Yes	50 (100)
Method of checking thawing time	
Watch	11 (22)
Manual/mental counting	38 (76)
Others	1 (2)
Thawing time practiced (seconds)	
1-10	28 (56)
11-20	14 (28)
21 and above 30	4 (8)
Instrument used to cut straw	
Cutter	27 (54)
Scissors	29 (58)
AI straw orientation during cutting	
Angled	12 (24)
Straight	38 (76)
Use of plastic gloves during rectal palpation	
No	1 (2)
Yes	49 (98)
Reusing of gloves	
No	26 (52)
Yes	24 (48)
Use of lubricant	
No	8 (16)
Yes	41 (82)
Lubricant used	
Soap	33 (66)
Manure	25 (50)
KY Jelly	1 (2)
Others	1 (2)
Site of usual semen deposition	
Mid-cervix	9 (18)
Entrance	4 (8)
Body of uterus	43 (86)
Deposition after ideal time elapsed	
Mid-cervix	22 (44)
Entrance	17 (34)
Body of uterus	12 (24)
Vaginal canal	0 (0)
Usual time of insemination	
Morning	22 (44)
Afternoon	9 (18)
Both times	19 (38)
Frequency of insemination	
Once	1 (2)
Twice	8 (16)
Thrice	37 (74)
Confirmation of pregnancy	
No	2 (4)
Yes	48 (96)
Performance of pregnancy diagnosis post-AI (months)	
1	2 (4)
2	4 (8)
3	32 (64)
4	6 (12)
5 or more	2 (4)
Estrus synchronization	
No	0 (0)
Sometimes	43 (86)
Always	4 (8)
Education of farmers	
No	0 (0)
Yes	50 (100)

AI=Artificial insemination

### AI success rates

The operation year 2011 had the highest recorded success rate (Table-3). High number of trainings were reportedly conducted during this year (personal communication), which might have an influence on the performance. The lowest observed success rate was in 2015, which may be due to lack of an updated reported data on calf drops. A high success rate (64.4%) was observed in Biliran in 2012 but may be misleading due to the low number of recorded AIs performed (45 inseminations only) during the year. A decrease in the success rates after 2011 was observed, for most locations in 2013. The decrease in success rates may have been caused by Super Typhoon Haiyan, which caused major damage to Eastern Visayas. A slight increase in success rates can be observed in the year 2014, indicating recovery from the aftermath of the typhoon. However, the computed success rates were found lower than the national performance (40%) [15].

**Table-3:** AI success rates (%) of selected areas in Eastern Visayas, Philippines (2011-15).

Location	2011	2012	2013	2014	2015
Leyte	11.4	8.8	8.1	9.9	3.0
Southern Leyte	17.4	4.7	8.9	9.7	0.9
Biliran	13.5	64.4	-	7.1	1.1
Western Samar	10.8	-	10.9	10.3	1.8
Northern Samar	6.2	6.3	1.7	1.0	-

AI=Artificial insemination

### Statistical analyses

A significant difference was found between AI success and civil status of AITs for most years except 2012 (Table-4). This study showed that married technicians had higher success rates than single or widowed AITs. While further study may be needed to clarify the role of civil status in AI performance, married technicians may be more patient and with better interpersonal skills, which were found associated with good inseminators [10]. A significant correlation was also found between the average number of AI per year and AI success. Technicians having an average of more than 100 AIs had higher success rates than those who had below 100 inseminations per year. This is contrary to the findings of Cembrowics [16] where the number of inseminations did not affect the success of AI. However, a high number of inseminations can be associated with better familiarity of the reproductive system [11] and higher confidence of the technician [17] leading to higher success rates.

**Table-4:** Results of statistical analyses with significant results (p value).

Parameter	Year				

	2011	2012	2013	2014	2015
Profile					
Civil status	0.050*	0.922	0.047*	0.034*	0.039*
Average AI per year	0.085	0.217	0.766	0.858	0.008**
Number of trainings attended	0.025*	0.389	0.047*	0.072	0.161
Training at PCC Ubay	0.007**	0.783	0.000**	0.010**	0.000**
Practice/practice					
Checking heat by soft cervix	0.056	0.399	0.208	0.009**	0.253
Checking heat by rectal palpation	0.613	0.41	0.008**	0.004**	0.504
Checking heat by reddish coloring of vagina	0.014*	0.492	0.752	0.767	0.292
Checking heat by the amount of vaginal discharge	0.046*	0.353	0.115	0.012*	0.713
Checking of thawing time using watch	0.174	0.234	0.517	0.483	0.018*
Cutting AI straw using cutter	0.017*	0.258	0.15	0.107	0.013*
Cutting AI straw with an angled orientation	0.377	0.48	0.225	0.009**	0.283
Semen deposition at the entrance of the cervix	0.154	0.81	0.247	0.462	0.000***
Semen deposition at the uterine body	0.019*	0.73	0.63	0.265	0.523
Deposition at mid-cervix after standard time elapsed	0.399	0.145	0.035*	0.059	0.884
Deposition at entrance after standard time elapsed	0.033*	0.243	0.286	0.614	0.013*
Deposition at uterine body after standard time elapsed	0.222	0.726	0.26	0.004**	0.005**

* Significant,

**/***Highly significant. AI=Artificial insemination

The study also found a significant association between the number of trainings attended and AI success rate. However, this could be considered a confounder because all AITs were required to attend at least one training before practicing AI. Studies have shown that participation in refresher courses can lead to higher AI success rates [13,14,17,18]. Moreover, statistical significance was found between training in PCC Ubay and AI success rate. Results showed that AITs who attended training in PCC Ubay have lower success rates than those who attended training in VSU or in Luzon. Since training of AITs has been shown to be very vital in maintaining a satisfactory breeding efficiency [17], further studies are needed to evaluate this observation because training in PCC Ubay is longer in duration than those conducted in PCC at VSU.

For the AI practices employed, the analysis revealed that checking of heat through evaluation of soft cervix, reddish coloring of vagina, amount of discharge and by rectal palpation, and checking of thawing time using watch were significant. Checking and monitoring of heat and thawing time have been shown to be vital to the health and breeding efficiency of animals [19-21]. On the other hand, cutting the AI straw using a cutter with an angle orientation was also found statistically significant. Duponte [22] explained that cutting the straw straight across prevents back flushing of semen into the insemination gun. Moreover, semen deposition at the entrance of the cervix, uterine body, and at mid-cervix, and at the entrance of cervix and uterine body after standard time elapsed were also found significant. Semen deposition site is shown to influence calves’ sex [23], but another study noted that semen deposition sites have no effect and that sperm concentration appeared to be more significant in the success of conception [24].

## Conclusion

Technician profile factors, including civil status, average AI per year, the number of trainings attended, and training may have an influence on the success of AI. For the practices, checking of soft cervix, rectal palpation, reddish coloring of vagina, amount of discharge, thawing temperature check by hand, thawing time check by watch, cutting straw by cutter and at an angle, semen deposition at entrance, semen deposition at uterus body, and deposition at mid-cervix may also influence the success of AI. From 2011 to 2015, AI success rates in Leyte, Samar, and Biliran were below the 40% national performance. The AITs, farmers, and LGU must exert more effort to improving AI performance. Although technician’s profile and practices can be critical to the success of AI, other factors including social and logistics may also be significant. It is recommended that AITs should attempt to perform more AIs per year. Moreover, a nationwide study may be conducted to assess and evaluate the profile, practices, and performance of AITs to improve national AI performance rate.

## Authors’ Contributions

APY and RHDY contributed equally to the study. APY and RHDY conceptualized the study, and analyzed and wrote the manuscript. MOC, LVM, JVA, ESN, ESN, JTR and IFML contributed in the data collection and giving valuable inputs to the manuscript. All authors have read and approved the final manuscript.

## References

[1] Khalifa T, Rekkas C, Samartzi F, Lymberopoulos A, Kousenidis K, Dovenski T (2014). Highlights on artificial insemination (AI) technology in the pigs. Maced. Vet. Rev.

[2] Lima F. S, de Vries A, Risco C. A, Santos J. E. P, Thatcher W. W (2010). Economic comparison of natural service and timed artificial insemination breeding programs in dairy cattle. J. Dairy Sci.

[3] Rehmana F. U, Zhaoa C, Shaha M. A, Qureshib M. S, Wanga X (2013). Semen extenders and artificial insemination in ruminants. Veterinaria.

[4] Inchaisri C, Jorritsma R, Vernooij J. C. M, Vos P. L. A, Van der Weijden G. C, Hogeveen H (2011). Cow effects and estimation of success of first and following inseminations in Dutch dairy cows. Reprod. Domest. Anim.

[5] Duran P, Corpuz H. K, Gaspar D. C, Misola C. M, Munar M, Hufana-Duran D (2015). Non-invasive clinical diagnosis of estrus for AI synchronization using vaginal cytology in three bubaline breeds in the Philippines. Res. J. Pharm. Biol. Chem. Sci.

[6] Cruz L. C (2010). Recent developments in the buffalo industry of Asia. Rev. Vet.

[7] Duran D, Duran P, Purohit G. N (2015). Advanced reproductive technologies in water buffalo. Bubaline Theriogeneology.

[8] Chaikhun T, Hengtrakunsin R, De Rensis F, Techakumphu M, Suadsong S (2012). Reproductive and dairy performances of Thai swamp buffaloes under intensive farm management. Thai. J. Vet. Med.

[9] Perry G. A, Dalton J. C, Geary T. W (2010). Management factors influencing fertility in synchronized and natural breeding programs. Proceedings, Applied Reproductive Strategies in Beef Cattle January 28-29 2010.

[10] Russi L, Costa-e-Silva L, Zuccari C, Recalde C (2010). Human resources in artificial insemination of beef cattle: Profile of managers and inseminators. Rev. Bras. Zootec.

[11] Singh J, Nanda A (2007). Interventions for improving the fertility of crossbred cows subjected to artificial insemination under field conditions. Improving the Reproductive Management of Dairy Cattle Subjected to Artificial Insemination.

[12] Everett R, Bean B (1986). Semen fertility - An evaluation system for artificial insemination sires, technicians, herds, and systemic fixed effects. J. Dairy Sci.

[13] Akhtar T, Lodhi L, Khanum S, Rashad M, Hussain M (2007). Improving reproductive efficiency in an artificial insemination programme through early non-pregnancy diagnosis, management and training. Improving the Reproductive Management of Dairy Cattle Subjected to Artificial Insemination.

[14] Boettcher P, Perera B (2007). Improving the reproductive management of smallholder dairy cattle and the effectiveness of artificial insemination: As summary. Improving the Reproductive Management of Dairy Cattle Subjected to Artificial Insemination.

[15] Roque A (2011). People Find Their Calling in Artificial Insemination.

[16] Cembrowics H (1964). Efficiency of inseminators Proceeding 5^th^. International Congress Animal Reproduction Artificial Insemination.

[17] Santolaria P, Lopez-Gatius F, Sanchez-Nadal J. A, Yaniz J (2012). Relationships between body weight and milk yield during the early postpartum period and bull and technician and the reproductive performance of high producing dairy cows. J. Reprod. Dev.

[18] Berry D. P, Evans R. D, Mc Parland S (2011). Evaluation of bull fertility in dairy and beef cattle using cow field data. Theriogenology.

[19] Aungier S. P. M, Roche J. F, Sheehy M, Crowe M. A (2012). Effects of management and health on the use of activity monitoring for estrus detection in dairy cows. J. Dairy Sci.

[20] Roca J, Parrilla I, Rodriguez-Martinez H, Gil M. A, Cuello C, Vazquez J. M, Martinez E. A (2011). Approaches towards efficient use of boar semen in the pig industry. Reprod. Domest. Anim.

[21] Oliviera L. Z, Arruda R. P, de Andrade A. F. C, Santos R. M, Beletti M. E, Peres R. F. G, Martins J. P. N, Hossepian de Lima V. F. M (2012). Effect of sequence of insemination after simultaneous thawing of multiple semen straws on conception rate to timed AI in suckled multiparous Nelore cows. Theriogenology.

[22] DuPonte M (2007). Proper Semen Handling During an Artificial Insemination Program. Cooperative Extension Service, College of Tropical Agriculture and Human Resources, University of Hawai’i at Mānoa.

[23] Zobel R, Gereš D, Pipal I, Buić V, Gračner D, Tkalcic S (2011). Influence of the semen deposition site on the calves sex ratio in simmental dairy cattle. Reprod. Domest. Anim.

[24] Foote R. H, Cole H. H, Cupps P. T (2013). Physiological aspects of artificial insemination. Reproduction in Domestic Animals.

